# Risk factors for postoperative delirium in orthopaedic hip surgery patients: a database review

**DOI:** 10.1186/s12891-024-07174-x

**Published:** 2024-01-17

**Authors:** Kylie T. Callan, Megan Donnelly, Brandon Lung, Maddison McLellan, Ryan DiGiovanni, William McMaster, Steven Yang, Russell Stitzlein

**Affiliations:** 1 University of California Irvine School of Medicine, Irvine, CA USA; 2 New York University Langone Medical Center, New York, NY USA; 3 University of California Irvine Health, Orange, CA USA

**Keywords:** Delirium, Total hip arthroplasty, Hip fracture, Osteoarthritis

## Abstract

**Background:**

Postoperative delirium is a common problem affecting admitted patients that decreases patient satisfaction and increases the cost and complexity of care. The purpose of this study was to use the American College of Surgeons National Surgical Quality Improvement Program (ACS-NSQIP) database to compare rates and risk factors of postoperative delirium for total hip arthroplasty (THA) and hemiarthroplasty patients indicated for osteoarthritis or proximal femur fracture.

**Methods:**

The 2021 NSQIP database was queried for patients using Current Procedural Terminology (CPT) codes for THA and hemiarthroplasty and ICD-10 codes for osteoarthritis or proximal femur fracture. Demographic, past medical history, preoperative labs, and functional status data were recorded. Procedural data were also collected. Finally, postoperative outcomes and complications were reviewed.

**Results:**

Overall, 16% of patients had postoperative delirium. Delirium patients were older on average (82.4 years vs. 80.7 years, *p* < 0.001), had a lower BMI (19.5 vs. 24.8, *p* < 0.001), were more likely to have a history of dementia (54.6% vs. 13.6%, *p* < 0.001), were less likely to have an independent functional status (*p* < 0.001) or live alone (*p* < 0.001), and were more likely to have sustained a recent fall (*p* < 0.001). Delirium patients were more likely to be hyponatremic or hypernatremic (*p* = 0.002), anemic (*p* < 0.001), and severely dehydrated (*p* < 0.001), among other lab abnormalities. Delirium patients were also more likely to experience additional postoperative complications, including pneumonia, pulmonary embolism, urinary tract infection, stroke, cardiac arrest, sepsis, and unplanned reoperation and readmission after discharge (all *p* < 0.05).

**Conclusions:**

In this study, factors associated with postoperative delirium in patients undergoing hemiarthroplasty and THA were identified, including older age, lower BMI, certain medical conditions, decreased functional status, certain lab abnormalities, and postoperative complications. These findings can be used by clinicians to better inform care and to determine when orthopaedic joint replacement patients may be at an increased risk for postoperative delirium.

## Background

Postoperative delirium is one of the most common complications affecting patients after surgery, with one study observing a rate of postoperative delirium of 24% [1]. Some risk factors suggested for developing postoperative delirium across surgical subspecialties include older age, nursing home residency, pre-existing cognitive impairment, psychiatric disorders, low albumin, and higher ASA score, among others [2]. In hip fracture patients specifically, older age, male sex, higher ASA class, lower BMI, functional dependence, smoking, hypertension, and dementia were all considered risk factors, in addition to others [3]. Delayed surgery and deeper anesthesia (measured using bispectral index [BIS] values on EEG, with deeper anesthesia having a target BIS reading of 35 and lighter anesthesia having a target BIS reading of 50) may also increase the risk of postoperative delirium [4]. However, in a randomized control trial of older patients undergoing hip fracture repair, regional and general anesthesia showed no differences in rates of postoperative delirium [5]. In patients with hip fractures, total hip arthroplasty was associated with higher rates of postoperative delirium [6]. A meta-analysis examining total joint patients found that additional factors may include length of surgery, length of hospital stay, and benzodiazepine use [7]. Opioids and ketamine intraoperatively may also increase risk [8].

Postoperative delirium can decrease patient satisfaction and increase the cost and complexity of care. Postoperative delirium increases the cost for both the patient and the facility, including longer hospital length of stay, 6.0 days vs 4.6 days [9]. Direct and indirect technical costs, routine nursing costs, pathology costs, medication costs, and consultation costs to medicine, psychiatry, and neurology were all increased for delirium patients [9].

One option for reducing rates of postoperative delirium is optimizing sleep quantity and quality. One meta-analysis of older adults found that the sleep aid melatonin was effective in decreasing rates of postoperative delirium, but the more-potent ramelteon did not have the same effect [10, 11]. Nursing and individualization of care interventions may also be effective. Two randomized control trials that studied postoperative delirium-prevention protocols found that they significantly decreased rates of postoperative delirium and increased sleep quality [12, 13]. It has also been suggested that avoiding use of benzodiazepines and adequately controlling pain may be beneficial [14]. Identification of preoperative risk factors would allow for the use of prevention techniques in populations at risk.

The purpose of this study was to use the American College of Surgeons National Surgical Quality Improvement Program (ACS-NSQIP) database to compare rates of postoperative delirium for total hip arthroplasty (THA) patients indicated for osteoarthritis, total hip arthroplasty patients indicated for proximal femur fracture, and hemiarthroplasty patients indicated for proximal femur fracture. Additionally, we sought to evaluate which preoperative characteristics put individuals at greater risk for postoperative delirium to better inform care and determine situations in which interventions may be appropriate. The hypotheses were (1) that factors such as increased age and decreased functional status would increase the rates of postoperative delirium and (2) the patients indicated for proximal femur fracture would have a higher rate of postoperative delirium. The intention was that this data could help determine non-remedial versus remedial comorbidities in patients that suffered postoperative delirium, with the hope being that some remedial comorbidities may be altered even in the short term of an acute femur fracture and identifying these may help shape clinical decision-making and allow clinicians to be more vigilant in expecting, managing, and preventing postoperative delirium in their patients.

## Methods

**Fig. 1:**
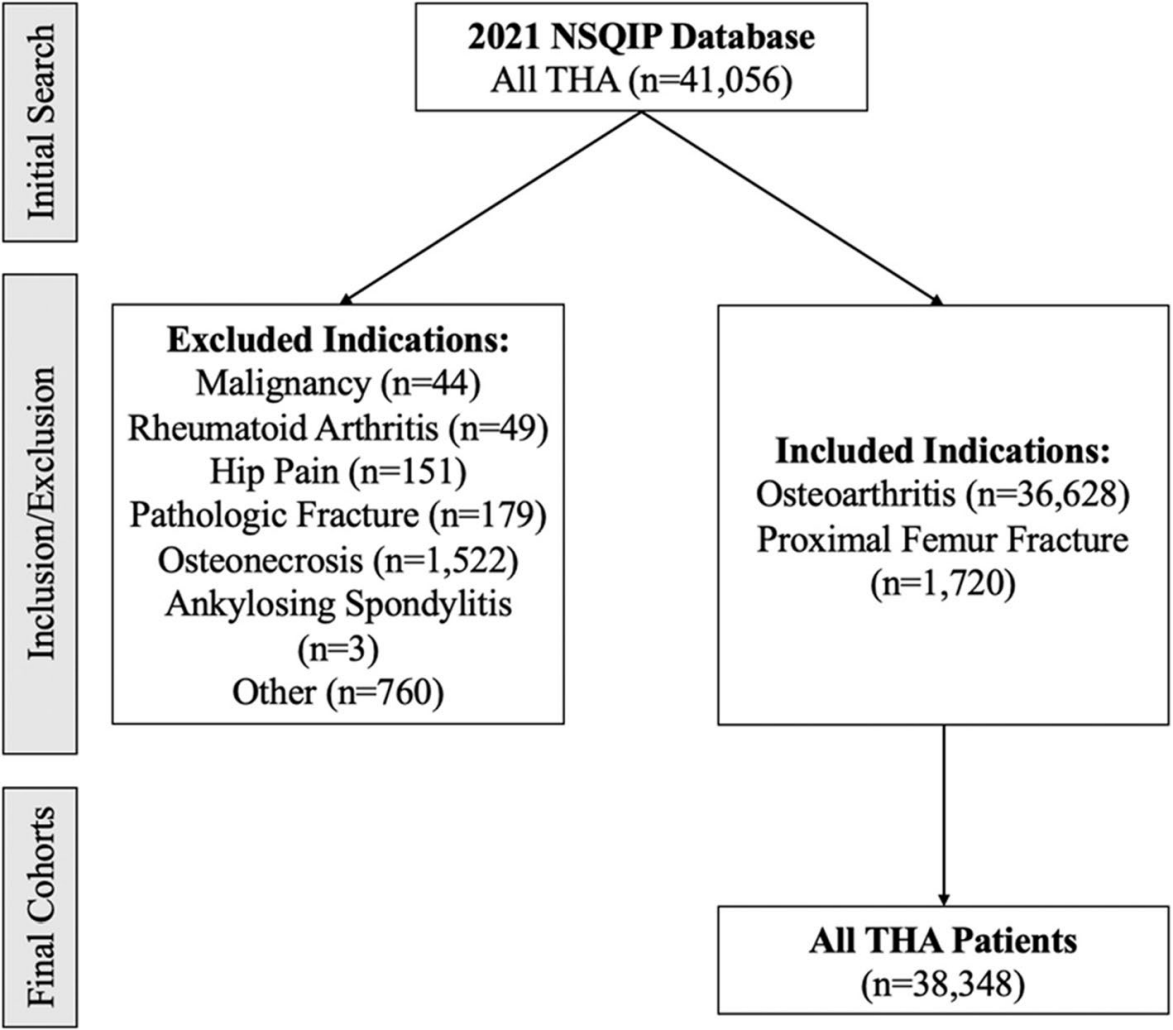
Inclusion criteria flowchart

This study was performed retrospectively using a validated, de-identified, and publicly available national database, and, as such, the study was considered exempt from the Institutional Review Board. No informed consent was required, and no funding was provided. Within the 2021 NSQIP database, subjects to be included in this study were identified using the Current Procedural Terminology (CPT) codes 27,130 for THA and 27,236 for hemiarthroplasty. This year of data was selected for inclusion in the study, as this was the first year within which information of postoperative delirium screening was collected. Data was only included for analysis for the THA group if the ICD-10 codes for indications included “M16” for osteoarthritis or “S72.0” for proximal femur fracture, and for the hemiarthroplasty patients if they had an indication of “S72.0” for proximal femur fracture. Contrarily, subjects were excluded from this study if they were < 18 years of age and if they underwent THA or hemiarthroplasty for other indications, detailed in Fig. 1.

Demographic data, such as patient sex, age, body mass index (BMI), race, and ethnicity, were recorded. Past medical history data, including dementia, diabetes, smoking status, baseline functional status (level of dependence for activities of daily living [ADLs] in 30 days prior to surgery), ventilator dependency, chronic obstructive pulmonary disease (COPD), ascites, congestive heart failure (CHF), hypertension requiring medication, dialysis, disseminated cancer, steroid/immunosuppressive therapy, bleeding disorder, requiring a preoperative packed red blood cell (pRBC) transfusion within 72 h before surgery, and sepsis, were also collected. Within the 2021 NSQIP, we were also able to collect data on if the patient lives alone, if they sustained a fall within 6 months of hospitalization, mortality and morbidity probability, and American Society of Anesthesiologists (ASA) class. With regards to surgical data, we recorded procedure type, indication, anesthesia type and operative time in minutes. Preoperative laboratory values were also collected and compared to normal ranges. Finally, postoperative outcomes and complications were also incorporated into our analyses, with an emphasis on postoperative delirium as our primary outcome.

Statistical analysis was performed for categorical variables using Chi-squared and Fischer’s exact test to compare preoperative characteristics and distribution of postoperative complications between patients with delirium and without delirium in the postoperative time period. Bivariate and multivariate logistic regression was performed to identify risk factors for delirium, including procedure type and indication. For the multivariate model, stepwise logistic regression was utilized to ensure incorporation of significant risk factors into the final analysis. Statistics were performed on IBM SPSS Statistics, Version 26 (IBM Corp., Armonk, NY). Significance was defined as *p* < 0.05.

## Results

A total of 7,361 subjects were identified for inclusion in this study and, of these, 16% had postoperative delirium as identified in the NSQIP chart screening. The distribution of sex, race and ethnicity did not vary between delirium and no delirium cohorts (all *p* > 0.05). Contrarily, delirium patients were found to be older on average (delirium: 82.4 years vs. no delirium: 80.7 years, *p* < 0.001) and had a lower BMI (delirium: 19.5 vs. no delirium: 24.8, *p* < 0.001). In terms of past medical history, delirium patients were more likely to have been diagnosed with dementia (delirium: 54.6% vs. no delirium: 13.6%, *p* < 0.001), ventilator dependence (delirium: 0.3% vs. no delirium: 0.1%, *p* = 0.026), COPD (delirium: 8.7% vs. no delirium: 7%, *p* = 0.038), CHF (delirium: 11.2% vs. no delirium: 7.1%, *p* < 0.001), and a bleeding disorder (delirium: 12.1% vs. no delirium: 8%, *p* < 0.001). Delirium patients also tended to be on dialysis more (delirium: 1.7% vs. no delirium: 0.7%, *p* = 0.002). On the other hand, delirium patients were less likely to have an independent functional status (delirium: 63.1% vs no delirium: 87.8%, *p* < 0.001) and were less likely to live alone (delirium: 23.6% vs. no delirium: 30.1%, *p* < 0.001). 62% of delirium patients sustained a fall within the 6 months prior to admission compared to only 30.1% of no delirium patients (*p* < 0.001). The majority of delirium patients had undergone hemiarthroplasty (79.5%), followed by THA for osteoarthritis (14.6%) and then THA for proximal femur fracture (5.9%). This was compared to no delirium patients, who mostly had undergone THA for osteoarthritis (54.5%) (*p* < 0.001). ASA class and anesthesia type also varied between cohorts, while average operative time was lower for delirium patients (delirium: 79 min vs. no delirium: 83 min, *p* < 0.001) (Table 1).

**Table 1:** Demographics and medical history characteristics of patients

	Overall (*n* = 46,788)	Delirium (*n* = 1,175)	No Delirium (*n* = 6,186)	*p* (Delirium vs No Delirium)
Sex, n (%)				0.124
Male	20,031 (42.8)	420 (35.7)	2,068 (33.4)	
Female	26,757 (57.2)	755 (64.3)	4,118 (66.6)	
Age, mean ± SD	68.7 ± 11.9	82.4 ± 4.3	80.7 ± 4.1	** < 0.001**
BMI, mean ± SD	29.5 ± 6.4	19.5 ± 11.9	24.8 ± 9.6	** < 0.001**
Race, n (%)				0.977
White	30,356 (86.0)	543 (90.2)	3,530 (90.6)	
Black or African American	3,476 (9.8)	32 (5.3)	208 (5.3)	
Asian	887 (2.5)	20 (3.3)	117 (3.0)	
Other Race	597 (1.7)	7 (1.2)	43 (1.1)	
Hispanic Ethnicity, n (%)	1,854 (5.2)	29 (4.7)	133 (3.4)	0.100
Past Medical History, n (%)				
Dementia	2,574 (17.3)	642 (54.6)	852 (13.8)	** < 0.001**
Diabetes	6,165 (13.2)	181 (15.4)	839 (13.6)	0.094
Smoker	4,856 (10.4)	61 (5.2)	256 (4.1)	0.103
Independent Functional Status	43,861 (94.2)	729 (63.1)	5,373 (87.8)	** < 0.001**
Ventilator Dependent	17 (0.0)	4 (0.3)	4 (0.1)	**0.026**
COPD	2,209 (4.7)	102 (8.7)	431 (7.0)	**0.038**
Ascites	33 (0.1)	1 (0.1)	4 (0.1)	0.581
CHF	1,669 (3.6)	132 (11.2)	440 (7.1)	** < 0.001**
Hypertension Requiring Medications	26,017 (55.6)	787 (67.0)	4,227 (68.3)	0.362
Dialysis	235 (0.5)	20 (1.7)	43 (0.7)	**0.002**
Disseminated Cancer	353 (0.8)	16 (1.4)	69 (1.1)	0.457
Immunosuppressive Therapy	1,854 (4.0)	47 (4.0)	264 (4.3)	0.752
Bleeding Disorder	1,966 (4.2)	142 (12.1)	493 (8.0)	** < 0.001**
Preoperative pRBC Transfusion < 72 Hours Before Surgery	209 (0.4)	28 (2.4)	52 (0.8)	** < 0.001**
Presence of Sepsis				** < 0.001**
No Sepsis	45,329 (96.9)	1,037 (88.3)	5,795 (93.7)	
SIRS	1,389 (3.0)	128 (10.9)	374 (6.0)	
Sepsis	63 (0.1)	8 (0.7)	17 (0.3)	
Septic Shock	7 (0.0)	2 (0.2)	0 (0)	
Lives Alone, n (%)	3,857 (29.1)	227 (23.6)	1,671 (30.1)	** < 0.001**
Fall Within 6 Months, n (%)	5,092 (36.2)	701 (62.0)	1,977 (33.6)	** < 0.001**
Mortality Probability, mean ± SD	0.01 ± 0.04	0.06 ± 0.07	0.03 ± 0.05	** < 0.001**
Morbidity Probability, mean ± SD	0.04 ± 0.04	0.11 ± 0.06	0.07 ± 0.05	** < 0.001**
Procedure, n (%)				** < 0.001**
Hemiarthroplasty	8,440 (18.0)	934 (79.5)	2,577 (41.7)	
THA for Osteoarthritis	36,628 (78.3)	172 (14.6)	3,370 (54.5)	
THA for Proximal Femur Fracture	1,720 (3.7)	69 (5.9)	239 (3.9)	
ASA Class, n (%)				** < 0.001**
1	1,225 (2.6)	3 (0.3)	41 (0.7)	
2	20,522 (44.0)	108 (9.3)	1,686 (27.3)	
3	22,362 (47.9)	734 (63.1)	3,676 (59.6)	
4	2,574 (5.5)	319 (27.4)	769 (12.5)	
General Anesthesia, n (%)	20,165 (43.1)	641 (54.6)	2,541 (41.1)	** < 0.001**
Operative Time Minutes, mean ± SD	89.9 ± 37.2	79.0 ± 43.4	83.2 ± 34.5	** < 0.001**

With regards to preoperative laboratory values, delirium patients were more likely to be hyponatremic (delirium: 14.2% vs. no delirium: 13.2%) or hypernatremic (delirium: 0.5% vs. no delirium: 0.1%) (*p* = 0.002). Similarly, patients with delirium were more likely to have preoperative leukocytosis (delirium: 24.6% vs. no delirium: 11.9%, *p* < 0.001) and low platelets (delirium: 11.5% vs. no delirium: 7.1%, *p* < 0.001). Delirium patients were also more likely to be anemic (delirium: 50.4% vs. no delirium: 33.7%, *p* < 0.001), severely dehydrated (delirium: 34.3% vs. no delirium: 27.7%, *p* < 0.001), hypoalbuminemic (delirium: 36.6% vs. no delirium: 18.5%, *p* < 0.006) and have a high alkaline phosphatase (delirium: 6.1% vs. no delirium: 3.7%, *p* = 0.006). Average INR was higher for delirium patients (delirium: 1.13 vs. no delirium: 1.10, *p* < 0.001) (Table 2).

**Table 2:** Laboratory characteristics of delirium and no delirium patients

	Delirium (*n* = 1,175)	No Delirium (*n* = 6,186)	*p*
Sodium Level, n (%)			**0.002**
Hyponatremia	162 (14.2)	778 (13.2)	
Normal Sodium	976 (85.3)	5,109 (86.7)	
Hypernatremia	6 (0.5)	5 (0.1)	
Leukocytosis, n (%)	283 (24.6)	708 (11.9)	** < 0.001**
Low Platelets, n (%)	132 (11.5)	424 (7.1)	** < 0.001**
Anemia, n (%)			** < 0.001**
No Anemia	572 (49.6)	3,946 (66.3)	
Mild Anemia	336 (29.1)	1,290 (21.7)	
Moderate/Severe Anemia	246 (21.3)	714 (12.0)	
Dehydration, n (%)			** < 0.001**
Not Dehydrated	420 (41.1)	2,310 (44.8)	
Moderately Dehydrated	251 (24.6)	1,418 (27.5)	
Severely Dehydrated	351 (34.3)	1,430 (27.7)	
Hypoalbuminemia, n (%)	260 (36.6)	603 (18.5)	** < 0.001**
High Alkaline Phosphatase, n (%)	39 (6.1)	103 (3.7)	**0.006**
PTT, mean ± SD	30.0 ± 7.1	30.1 ± 6.8	0.807
INR, mean ± SD	1.13 ± 0.22	1.10 ± 0.25	** < 0.001**

Along with the complication of delirium, delirium patients were more likely to experience postoperative pneumonia, unplanned reintubation, pulmonary embolism, failure to wean off the ventilator, urinary tract infection, stroke, cardiac arrest, myocardial infarction, bleeding requiring transfusion, sepsis, septic shock, unplanned reoperation and readmission after discharge (all *p* < 0.05) (Table 3).

**Table 3:** Postoperative complications of delirium and no delirium patients

	Delirium (*n* = 1,175)	No Delirium (*n* = 6,186)	*p*
Superficial SSI, n (%)	16 (1.4)	78 (1.3)	0.777
Deep SSI, n (%)	2 (0.2)	9 (0.1)	0.691
Organ Space SSI, n (%)	7 (0.6)	28 (0.5)	0.489
Wound Disruption, n (%)	1 (0.1)	12 (0.2)	0.706
Pneumonia, n (%)	86 (7.3)	97 (1.6)	** < 0.001**
Unplanned Reintubation, n (%)	24 (2.0)	19 (0.3)	** < 0.001**
Pulmonary Embolism, n (%)	23 (2.0)	51 (0.8)	**0.001**
Failure to Wean off Vent, n (%)	12 (1.0)	7 (0.1)	** < 0.001**
Renal Failure, n (%)	0 (0)	6 (0.1)	0.598
UTI, n (%)	76 (6.5)	145 (2.3)	** < 0.001**
CVA/Stroke, n (%)	25 (2.1)	21 (0.3)	** < 0.001**
Cardiac Arrest, n (%)	11 (0.9)	24 (0.4)	**0.019**
MI, n (%)	58 (4.9)	86 (1.4)	** < 0.001**
Bleeding Requiring Transfusion, n (%)	134 (11.4)	377 (6.1)	** < 0.001**
DVT, n (%)	14 (1.2)	43 (0.7)	0.099
Sepsis, n (%)	18 (1.5)	28 (0.5)	** < 0.001**
Septic Shock, n (%)	12 (1.0)	13 (0.2)	** < 0.001**
Reoperation, n (%)	38 (3.2)	120 (1.9)	**0.005**
Unplanned Readmission, n (%)	124 (10.6)	337 (5.4)	** < 0.001**

On bivariate analysis comparing procedure type and risk of delirium, hemiarthroplasty (OR: 7.101, 95% CI: [5.988, 8.422], *p* < 0.001) and THA for proximal femur fracture (OR: 5.657, 95% CI: [4.155, 7.710], *p* < 0.001) were associated with increased delirium risk compared to THA for osteoarthritis (Table 4). This increased risk held true in the multivariate model controlling for other preoperative risk factors (Table 4).

**Table 4:** Bivariate and multivariate regression associated with postoperative delirium

**Bivariate Regression of Procedure Type (Reference = THA for Osteoarthritis)**			
	OR	95% CI	*p*
Hemiarthroplasty	7.101	(5.988, 8.422)	** < 0.001**
THA for Proximal Femur Fracture	5.657	(4.155, 7.701)	** < 0.001**
**Multivariate Regression**			
	OR	95% CI	*p*
Hemiarthroplasty	2.297	(1.817, 2.902)	** < 0.001**
THA for Proximal Femur Fracture	2.777	(1.930, 3.994)	** < 0.001**
Fall in the Last 6 Months	0.928	(0.777, 1.109)	0.412
Dementia	4.484	(3.813, 5.272)	** < 0.001**
CHF	1.047	(0.821, 1.336)	0.710
Hypertension Requiring Medications	0.946	(0.809, 1.106)	0.487
Dialysis	1.241	(0.676, 2.277)	0.485
General Anesthesia	1.216	(1.045, 1.413)	**0.011**
Total Hospital Length of Stay Days	1.089	(1.074, 1.103)	** < 0.001**

When the patients were divided into groups based on indication, there were significant differences in demographics. Femur fracture patients were more likely to be female (65.3% vs 54.9%, *p* < 0.001), older (75.1 years vs 66.0 years, *p* < 0.001), have a lower BMI (21.7 vs 30.3, *p* < 0.001), have any medical comorbidities (all *p* < 0.001), have SIRS, sepsis, or septic shock (*p* < 0.001), have fallen in the previous six months (69.5% vs 6.1%, *p* < 0.001), have a higher ASA class (*p* < 0.001), and undergo general anesthesia (67.6% vs 36.3%, *p* < 0.001) compared to OA patients (Table 5). In terms of laboratory levels, proximal femur fracture patients were more likely than OA patients to have abnormal sodium levels (17.5% hyponatremia vs 4.3%, and 0.3% hypernatremia vs 0%, *p* < 0.001), have a leukocytosis (22.4% vs 1.7%, *p* < 0.001), have low platelets (11.7% vs 2.1%, *p* < 0.001), have anemia (*p* < 0.001), be dehydrated (*p* < 0.001), or be hypoalbuminemic (35.3% vs 2.3%, *p* < 0.001), among other laboratory abnormalities shown in Table 5.

**Table 5:** Demographics of proximal femur fracture vs OA patients

	Proximal Femur Fracture (*n* = 10,160)	Osteoarthritis (*n* = 36,628)	*p*
Sex, n (%)			** < 0.001**
Male	3,522 (34.7)	16,505 (45.1)	
Female	6,637 (65.3)	20,120 (54.9)	
Age, mean ± SD	75.1 ± 11.2	66.0 ± 10.5	** < 0.001**
BMI, mean ± SD	21.7 ± 10.4	30.3 ± 6.7	** < 0.001**
Race, n (%)			** < 0.001**
White	6,134 (88.4)	24,222 (85.4)	
Black or African American	438 (6.3)	3,038 (10.7)	
Asian	246 (3.5)	641 (2.3)	
Other Race	122 (1.8)	475 (1.7)	
Hispanic Ethnicity, n (%)	406 (5.7)	1,448 (5.1)	**0.031**
Past Medical History, n (%)			
Dementia	2,330 (34.0)	244 (3.0)	** < 0.001**
Diabetes	1,756 (17.3)	4,409 (12.0)	** < 0.001**
Smoker	1,243 (12.2)	3,613 (9.9)	** < 0.001**
Independent Functional Status	7,916 (78.8)	35,945 (98.4)	** < 0.001**
Ventilator Dependent	17 (0.2)	0 (0)	** < 0.001**
COPD	932 (9.2)	1,277 (3.5)	** < 0.001**
Ascites	29 (0.3)	4 (0)	** < 0.001**
CHF	920 (9.1)	749 (2.0)	** < 0.001**
Hypertension Requiring Medications	6,223 (61.3)	19,794 (54.0)	** < 0.001**
Dialysis	193 (1.9)	42 (0.1)	** < 0.001**
Disseminated Cancer	269 (2.6)	84 (0.2)	** < 0.001**
Immunosuppressive Therapy	526 (5.2)	1,328 (3.6)	** < 0.001**
Bleeding Disorder	1,322 (13.0)	644 (1.8)	** < 0.001**
Preoperative pRBC Transfusion < 72 Hours Before Surgery	198 (1.9)	11 (0)	** < 0.001**
Presence of Sepsis			** < 0.001**
No Sepsis	8,829 (87.0)	36,500 (99.7)	
SIRS	1,261 (12.4)	128 (0.3)	
Sepsis	63 (0.6)	0 (0)	
Septic Shock	7 (0.1)	0 (0)	
Lives Alone, n (%)	1,535 (27.6)	2,322 (30.2)	**0.001**
Fall Within 6 Months, n (%)	4,640 (69.5)	452 (6.1)	** < 0.001**
Mortality Probability, mean ± SD	0.045 ± 0.064	0.001 ± 0.002	** < 0.001**
Morbidity Probability, mean ± SD	0.095 ± 0.054	0.027 ± 0.012	** < 0.001**
Procedure, n (%)			** < 0.001**
Hemiarthroplasty	8,440 (83.1)	–	
THA	1,720 (16.9)	36,628 (100)	
ASA Class, n (%)			** < 0.001**
1	123 (1.2)	1,102 (3.0)	
2	1,977 (19.5)	18,545 (50.7)	
3	6,115 (60.5)	16,247 (44.4)	
4	1,898 (18.8)	676 (1.8)	
General Anesthesia, n (%)	6,868 (67.6)	13,297 (36.3)	** < 0.001**
Operative Time Minutes, mean ± SD	80.8 ± 39.9	92.4 ± 36.1	** < 0.001**
	Proximal Femur Fracture (*n* = 10,160)	Osteoarthritis (*n* = 36,628)	*p*
Sodium Level, n (%)			** < 0.001**
Hyponatremia	1,753 (17.5)	1,449 (4.3)	
Normal Sodium	8,254 (82.3)	31,938 (95.6)	
Hypernatremia	26 (0.3)	15 (0)	
Leukocytosis, n (%)	2,255 (22.4)	565 (1.7)	** < 0.001**
Low Platelets, n (%)	1,177 (11.7)	708 (2.1)	** < 0.001**
Anemia, n (%)			** < 0.001**
No Anemia	5,112 (50.8)	30,083 (87.6)	
Mild Anemia	2,767 (27.5)	3,535 (10.3)	
Moderate/Severe Anemia	2,185 (21.7)	720 (2.1)	
Dehydration, n (%)			** < 0.001**
Not Dehydrated	4,550 (48.4)	17,534 (57.4)	
Moderately Dehydrated	2,273 (24.2)	7,314 (23.9)	
Severely Dehydrated	2,572 (27.4)	5,696 (18.6)	
Hypoalbuminemia, n (%)	2,368 (35.3)	464 (2.3)	** < 0.001**
High Alkaline Phosphatase, n (%)	455 (7.3)	397 (2.3)	** < 0.001**
PTT, mean ± SD	29.8 ± 7.1	29.3 ± 5.5	** < 0.001**
INR, mean ± SD	1.1 ± 0.2	1.0 ± 0.2	** < 0.001**

Delirium rates were 26.9% for hemiarthroplasty for femur fracture, 22.4% for THA for femur fracture, and 4.8% for THA for OA (*p* < 0.001) (Table 6).

**Table 6:** Rates of Delirium and Multivariate Regression of Risk Factors for Delirium by Procedure and Indication

	Hemiarthroplasty for Proximal Femur Fracture	THA for Proximal Femur Fracture	THA for OA	*p*
Delirium, n (%)	1,007 (26.9)	69 (22.4)	174 (4.8)	** < 0.001**
**Hemiarthroplasty for Proximal Femur Fracture**				
	OR	95% CI	*p*	
Fall in the Last 6 Months	0.841	(0.701, 1.009)	0.062	
Dementia	4.362	(3.692, 5.155)	** < 0.001**	
CHF	1.149	(0.897, 1.471)	0.272	
Hypertension Requiring Medications	0.905	(0.761, 1.077)	0.260	
Dialysis	1.258	(0.701, 2.260)	0.442	
General Anesthesia	1.156	(0.980, 1.363)	0.086	
Total Hospital Length of Stay Days	1.075	(1.060, 1.090)	** < 0.001**	
**THA for Proximal Femur Fracture**				
	OR	95% CI	*p*	
Fall in the Last 6 Months	1.505	(0.856, 2.647)	0.156	
Dementia	6.133	(3.614, 10.406)	** < 0.001**	
CHF	0.913	(0.405, 2.058)	0.826	
Hypertension Requiring Medications	0.933	(0.652, 1.335)	0.703	
General Anesthesia	1.339	(0.946, 1.895)	0.100	
Total Hospital Length of Stay Days	1.204	(1.157, 1.252)	** < 0.001**	
**THA for OA**				
	OR	95% CI	*p*	
Fall in the Last 6 Months	1.505	(0.760, 2.982)	0.241	
Dementia	5.652	(2.918, 10.947)	** < 0.001**	
CHF	0.517	(0.145, 1.845)	0.309	
Hypertension Requiring Medications	1.353	(0.708, 2.584)	0.360	
General Anesthesia	0.764	(0.396, 1.471)	0.420	
Total Hospital Length of Stay Days	1.044	(0.971, 1.123)	0.241	

Multivariate regression analysis results showed history of dementia and hospital length of stay were associated with delirium in the femur fracture group, and history of dementia was associated with delirium in the THA for OA group (all *p* < 0.001). History of fall in the last six months, CHF, hypertension, dialysis, or general anesthesia were not significantly associated for separated groups (Table 6).

## Discussion

In this study, delirium patients were older, had lower BMI, were more likely to have medical conditions such as dementia, COPD, CHF, or a bleeding disorder, were more likely to be on dialysis, were less likely to be functionally independent or live alone, were more likely to have sustained a recent fall, were most likely to have undergone a hemiarthroplasty, and had shorter surgical times. They had lab abnormalities including abnormal sodium levels, leukocytosis, low platelets, anemia, severe dehydration, hypoalbuminemia, a high alkaline phosphatase, and an elevated INR. They were more likely to experience postoperative pneumonia, unplanned reintubation, pulmonary embolism, failure to wean off the ventilator, urinary tract infection, stroke, cardiac arrest, myocardial infarction, bleeding requiring transfusion, sepsis, septic shock, unplanned reoperation, and readmission after discharge. Hemiarthroplasty and THA for femur fracture were associated with higher rates of delirium than THA for osteoarthritis. Finally, when multivariate analysis was performed for the separate indications, history of dementia was associated with delirium in all groups and hospital length of stay was associated with delirium in both proximal femur fracture groups.

The demographic differences between the proximal femur fracture group and the OA group are in line with what was expected. Patients who sustain proximal femur fractures and require non-elective surgery are more likely to be more ill, as evidenced by higher rates of medical comorbidities and higher ASA scores, and patients who undergo elective THA for OA are more likely to have higher BMI and be younger [15, 16].

The overall rate of delirium was 16% in this study, with rates of 26.9% for hemiarthroplasty for femur fracture, 22.4% for THA for femur fracture, and 4.8% for THA for OA. This rate of postoperative delirium after hemiarthroplasty for proximal femur fracture is consistent with what has been seen in some other studies, with two studies seeing rates of 26% and 30.9% of elderly femoral neck fracture patients experiencing delirium after hemiarthroplasty [17, 18]. The rate of delirium seen in elective THA patients is also in line with what many other studies have seen. Although some studies cite lower rates such as 0.7–2.2%, others saw rates of 8.6–18.4% in elective THA patients, and a review paper found a range of rates of 0–48% in papers examining elective total hip and total knee patients, with a median of 14.8% [19–23].

It has been well established that advanced age puts a patient at higher risk for postoperative delirium, which is in line with what this study found [8, 21, 24, 25]. One systematic review of patients undergoing hip and knee arthroplasty found that advanced age increased the risk of postoperative delirium with an odds ratio of 3.81. [26] Another observational cohort study found that each additional year of age had an odds ratio of 1.1 of developing postoperative delirium in elective arthroplasty patients [27]. Poeran and colleagues examined patients undergoing hip fracture repair and found that not only did the rate of postoperative delirium increase as age increased, but that there was no clear plateau in rate [28]. Therefore, this finding was in line with the hypotheses as well as the current state of the literature.

In this study, patients with lower BMI were at higher risk for postoperative complications. However, the literature on outcomes based on BMI is mixed. For example, one systematic review of patients undergoing hip and knee arthroplasty found no change in risk based on BMI [26]. However, another retrospective study examining hip fracture patients showed that decreased BMI had an increased risk of postoperative delirium, similar to what this study saw [29]. Furthermore, there was another retrospective study examining hip fracture patients that found that higher BMI was associated with increased rates of postoperative delirium [30]. It could be argued that both decreased and increased BMI lower a patient’s overall health status and could contribute to poor outcomes, as increased BMI could be associated with other health problems and decreased BMI could be a sign of frailty.

It is well established that a history of dementia increases a patient’s odds of developing postoperative delirium. This was true for the patients in this study as well, both when compared as a large group and when subdivided into groups by indication. A systematic review by Wu of hip fracture patients found that patients with dementia had a relative risk of 2.6 of developing postoperative delirium [3]. A retrospective cohort study of elderly hip fracture patients saw a rate of postoperative delirium of 58% in patients with dementia compared to 35% overall [31]. In terms of functional dependence, the finding of this study is consistent with other studies. It has been shown that patients with preoperative functional dependence have an increased risk of developing postoperative delirium, and Wu and colleagues found that functionally dependent patients had a relative risk of 1.52 of developing postoperative delirium [3, 21, 31].

There is more limited data available on preoperative laboratory markers associated with postoperative delirium, such as the abnormal sodium levels, leukocytosis, low platelets, anemia, severe dehydration, hypoalbuminemia, a high alkaline phosphatase, and an elevated INR seen in the delirium patients in this study. A study by Zhang et al. found that severe preoperative hypoalbuminemia was associated with an increased risk of postoperative delirium, but not mild or moderate hypoalbuminemia [32]. Chu and colleagues found delirium to be associated with hypoalbuminemia in hip fracture patients, but found no statistically significant difference in rates of delirium in anemic patients [29]. However, a 2011 cohort study in hip fracture patients showed increased rates of delirium in anemic patients [33]. A meta-analysis of geriatric hip fracture patients found that both hypo- and hypernatremia increased risk of postoperative delirium, which is consistent with this study’s findings [6].

In this study, patients with delirium were more likely to experience postoperative pneumonia, unplanned reintubation, pulmonary embolism, failure to wean off the ventilator, urinary tract infection, stroke, cardiac arrest, myocardial infarction, bleeding requiring transfusion, sepsis, septic shock, unplanned reoperation, and readmission after discharge. This is somewhat consistent with the literature, as delirium has been associated with longer length of stay, higher rate of overall complications, and higher six-month mortality in elderly hip fracture patients [31]. In terms of other postoperative complications, in a retrospective cohort study of elderly hip fracture patients in Korea, postoperative delirium was a risk factor for developing postoperative pneumonia [34]. Elderly hip fracture patients with postoperative delirium have also been shown to be at an increased risk of contralateral hip fracture within six months, pulmonary complications, neurological complications, renal function disorders, urinary tract infections (UTIs), bladder retention, hemodynamic instability, and cerebral vascular accidents (CVA) or transient ischemic attacks (TIA) [31]. Another retrospective database analysis examining TKA patients found that postoperative delirium was associated with acute renal failure, myocardial infarction, pneumonia, pulmonary embolism (PE), stroke, UTI, deep vein thrombosis (DVT), and sepsis [35]. In hip fracture patients over 60 years old, readmission and revision surgery rates were higher in those with postoperative delirium compared to those without postoperative delirium [36]. While some of these complications overlap with those seen in this study and some do not, some of these differences may be limited by the variables chosen by each study. Additionally, although many of these complications are associated with each other, it is difficult to discern whether certain complications put a patient at higher risk for other complications or if there may simply be factors in a patient’s medical history and operative course that decrease their baseline level of health and/or increase their risk for all postoperative complications. There are some studies that suggest that short-stem, cementless implants could be used to decrease the rates of complication in THA [37].

While this study saw that hemiarthroplasty and THA for fracture were associated with higher rates of delirium than THA for osteoarthritis, there is limited data directly examining the rates of postoperative delirium in different orthopaedic procedures. One study that examined rates of postoperative delirium in orthopaedic patients found no difference in rates following spine procedures, hip replacement, and fracture repair [38]. Another review found that orthopedic hip surgery patients were at an increased risk of developing postoperative delirium compared to other orthopedic surgical sites, such as knees or spine, and hypothesized this may be due to the larger blood losses, longer period of non-ambulation, and older patient age in hip surgeries [39]. In terms of operative time, the shorter operative time seen in the delirium group compared to the no-delirium group could be attributed to the fact that more delirium patients were undergoing hemiarthroplasty and more no-delirium patients were undergoing the more-complex THA.

This study does have limitations, and any findings should be interpreted in the context of these limitations. As it is a retrospective database study, the study uses data pooled from different practice settings in different parts of the country. Surgical protocols, follow-up timelines, and reporting conventions may therefore vary throughout the study population. As postoperative delirium was a new variable included in the database in 2021, all of the data only came from one year. Additionally, as with any database study, the study variables are limited by what individual surgeons report, and no further investigation into individual cases and postoperative complications may be pursued. All patients were selected for inclusion and exclusion based on CPT and ICD-10 codes and selection could not be more nuanced. Similarly, many complications were reported as a binary yes or no, and no consideration could be given to the severity of symptoms. Finally, postoperative complications were only reported through 30 days postoperatively.

## Conclusions

In terms of the first aim of the study, to compare rates of postoperative delirium for THA for osteoarthritis, THA for femur fracture, and hemiarthroplasty for femur fracture, the study determined that rates of delirium were higher in patients who had undergone hemiarthroplasty and THA for femur fracture as compared to THA for osteoarthritis. For the second aim of the study, to evaluate which preoperative characteristics put individuals at greater risk for postoperative delirium, it was observed that delirium was associated with older age, lower BMI, medical conditions including COPD, CHF, a bleeding disorder, or dialysis, were less likely to be functionally independent, were more likely to have sustained a recent fall, had lab abnormalities including hypo or hypernatremia, leukocytosis, low platelets, anemia, severe dehydration, hypoalbuminemia, a high alkaline phosphatase, and an elevated INR, and were more likely to suffer a large number of postoperative complications, including reoperation and readmission. These findings were consistent with the hypotheses that factors such as increased age and decreased functional status would increase the rates of postoperative delirium and that the patients indicated for proximal femur fracture would have a higher rate of postoperative delirium. Overall, this data helps clinicians consider which modifiable patient factors should be optimized prior to surgery to decrease rates of postoperative delirium, such as medical conditions, functional status, dehydration, anemia, or other laboratory abnormalities. Additionally, it provides a framework for determining which patients are at higher risk due to non-modifiable risk factors such as age, BMI, and medical comorbidities, allowing for closer observation and minimizing of risks.

## Data Availability

The data that support the findings of this study are available from the ACS-NSQIP database, but restrictions apply to the availability of these data, which were used with permission for the current study, and so are not publicly available. Data are however available from the authors upon reasonable request and with permission of NSQIP.

## Abbreviations

ACS-NSQIP: American College of Surgeons National Surgical Quality Improvement Program THA: Total hip arthroplasty
CPT: Current Procedural Terminology
BIS: Bispectral index
BMI: Body mass index
ADLs: Activities of daily living
COPD: Chronic obstructive pulmonary disease
CHF: Congestive heart failure
pRBC: Packed red blood cell
ASA: American Society of Anesthesiologists
UTI: Urinary tract infection
CVA: Cerebral vascular accident
TIA: Transient ischemic attack
PE: Pulmonary embolism
DVT: Deep vein thrombosis
